# Transfemoral Occlusion of Doubly Committed Subarterial Ventricular Septal Defect Using the Amplatzer Duct Occluder-II in Children

**DOI:** 10.3389/fcvm.2022.837847

**Published:** 2022-04-12

**Authors:** Changqing Tang, Kaiyu Zhou, Shuran Shao, Xiaoliang Liu, Yifei Li, Yimin Hua, Chuan Wang

**Affiliations:** ^1^Department of Pediatric Cardiology, West China Second University Hospital, Sichuan University, Chengdu, China; ^2^Department of Pediatric Cardiology, Children's Hospital of Soochow University, Suzhou, China; ^3^The Cardiac Development and Early Intervention Unit, West China Institute of Women and Children's Health, West China Second University Hospital, Sichuan University, Chengdu, China; ^4^Key Laboratory of Birth Defects and Related Diseases of Women and Children (Sichuan University), Ministry of Education Chengdu, Sichuan, China; ^5^Key Laboratory of Development and Diseases of Women and Children of Sichuan Province, West China Second University Hospital, Sichuan University, Chengdu, China

**Keywords:** doubly committed, subarterial ventricular septal defect, Amplatzer duct occluder-II, transfemoral closure, children

## Abstract

**Backgrounds:**

The traditional treatment of doubly committed subarterial ventricular septal defect (dcVSD) is open-heart surgery. This study aimed to evaluate the feasibility, safety, and outcome of transcatheter closure of small dcVSD using Amplatzer duct occluder-II (ADO-II) in children.

**Methods:**

Between January 2016 and April 2021, 24 children (17 male and 7 female patients) with small dcVSD who received transfemoral closure with ADO-II were enrolled retrospectively. All of their available clinical and follow-up data were evaluated.

**Results:**

The patients' median age was 3.2 years (1.6–12.6 years, 4.2 ± 3.1 years) and body weight was 13.3 kg (10.0–38.5 kg, 16.5 ± 7.7 kg). Left ventricular angiography showed that the median dcVSD size was 2.0 mm (1.5–3.5 mm, 2.1 ± 0.6 mm). The device was successfully implanted in 23 patients (95.8%), and one patient failed to be closed because of the underestimation of defect size due to preoperative aortic valve prolapse, with 16 patients by the antegrade approach and eight patients by retrograde approach. The diameters of the device used were 3/4, 4/4, and 5/4 mm. The median operative time was 40.0 min (20.0–75.0 min, 41.7 ± 13.7 min), and the median fluoroscopic time was 5.0 min (3.0–25.0 min, 6.8 ± 5.0 min). With a follow-up duration of 1+ to 45+ months, only 1 patient presented with new-onset mild aortic regurgitation (AR).

**Conclusion:**

Transfemoral closure of small dcVSD with ADO-II is technically feasible and safe in the selected children. However, the development or worsening of AR requires long-term follow-up.

## Introduction

Ventricular septal defects (VSDs), one of the most common congenital heart diseases with an incidence of ~40% (1), are mainly divided into a perimembranous, muscular, inlet, and doubly committed subarterial in accordance with the anatomical location (2). Doubly committed subarterial VSD (dcVSD) located just beneath the aortic and pulmonary valves is relatively common among the Asian and Far East populations accounting for up to 32% of all VSDs (2, 3). Since spontaneous closure of dcVSD is rare and subsequent complications, mainly aortic valve deformity, are relatively frequent and easily progressive, early intervention is generally recommended for dcVSD as soon as the diagnosis is confirmed.

Open-heart surgery advocated as the gold standard for the treatment of dcVSD is associated with complications due to cardiopulmonary bypass and sternotomy, especially in children (4). Recently, minimally invasive perventricular device closure using the asymmetric occluder has emerged as a potential treatment option with acceptable short- and mid-term follow-up results (5–7), suggesting the possibility of the device closure for dcVSD. Whereas, there are some undesirable adverse events related to perventricular device closure such as coronary artery injury, complicated bleeding, pleural effusion, pneumothorax, postoperative cardiac dysfunction, and wound infection.

To avoid the aforementioned complications, transfemoral occlusion for dcVSD using different types of devices has been attempted in several pilot studies. An asymmetric occluder (Shanghai Shape Memory Alloy Co, Ltd., Shanghai, China) was used in the study conducted by Chen et al., but the success rate was as low as 66% owing to several technical difficulties as a result of the hard profile of the asymmetric device and special location of dcVSD (8). Afterward, the Amplatzer duct occluder-I (ADO-I) (St. Jude Medical; Starway Medical Technology, Inc) was chosen in two studies conducted by Shyu et al. (9) and Huang et al. (10), respectively. Although the success rate was acceptable using ADO-I, the relatively high incidence of aortic regurgitation (AR) post-procedurally and during the follow-up was still the main issue of concern. In addition, both studies were limited by a small sample size and most of the enrolled patients were adults. Therefore, finding an ideal device for transfemoral closure of dcVSD, particularly in children, is urgently needed.

Currently, a softer occluder, the Amplatzer duct occulder-II (ADO-II) which has polyester material, has been successfully applied to close small perimembranous VSD (pmVSD) with the advantage of technical ease because of the device's softness and flexibility. It could be deployed retrogradely without the establishment of the arterial–venous loop and could be used in small children even younger than 1 year of age with less material consumption and a small delivery system. Moreover, it was supposed that the ADO-II could adapt to different shapes of defect with less influence on adjacent tissues, such as the conduction system and aortic valve (11–13). Collectively, the ADO-II may be beneficial to avoid the occurrence of the aforementioned problems in dcVSD closure. However, little is known about transfemoral occlusion of dcVSD using the ADO-II, especially in children (14). Therefore, this study describes our initial experience with transfemoral device closure for small dcVSD using the ADO-II in children with an evaluation of the device's feasibility, safety, and follow-up outcome.

## Methods

### Ethics Statements

Written informed consent was provided by guardians of all patients. This study was approved by the University Ethics Committee on Human Subjects at Sichuan University [No. 2015(010)].

### Study Design and Patients

**Figure 1:**
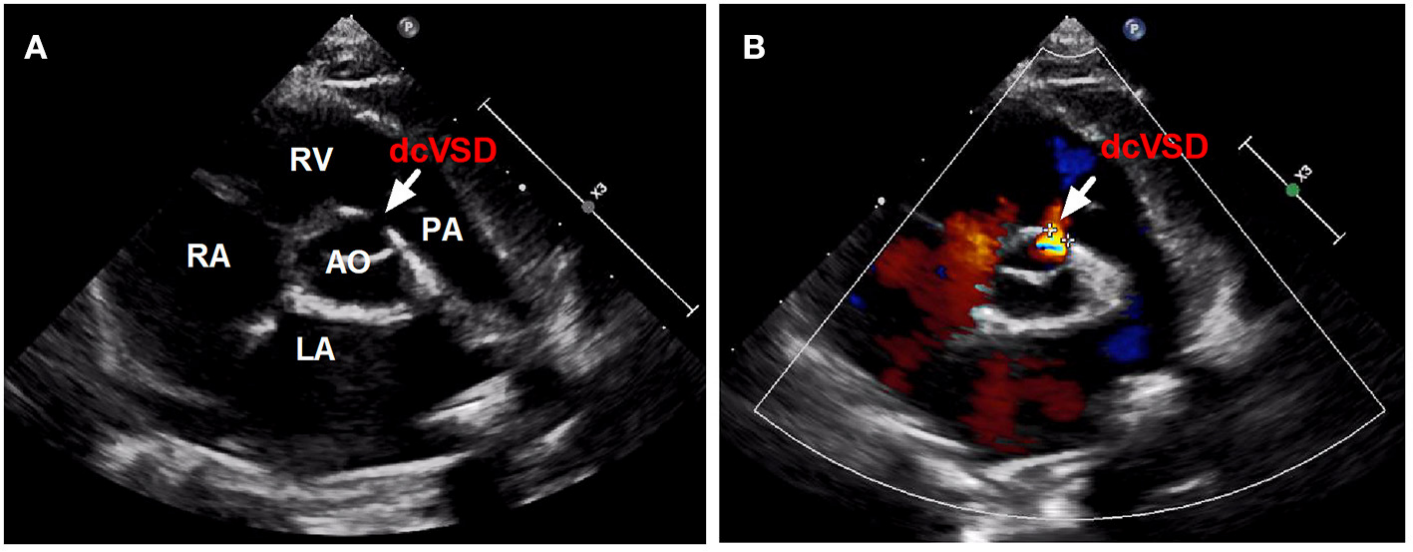
The confirmed diagnosis of dcVSD by TTE. Parasternal short-axis view **(A,B)** on TTE shows the dcVSD located at the 1-2 o'clock position (arrows). AO, aortic; LA, left atrium; RV, right ventricle; RA, right atrium; PA, pulmonary artery; dcVSD, doubly committed subarterial ventricular septal defect; TTE, transthoracic echocardiography.

Between January 2016 and April 2021, all children with dcVSD who underwent transfemoral closure using the ADO-II at our center were screened. The diagnosis of dcVSD was confirmed by transthoracic echocardiography (TTE), based on its position on an analog clock face in the short-axis parasternal view with the observation of dcVSD between the 1 and 2 o'clock directions (Figure 1) (15, 16). Aortic valve prolapse (AVP) was graded on a three-point scale: mild (buckling of the aortic cusp down the left ventricular [LV] outflow tract with minimal herniation into the VSD), moderate (prolapse of the cusp with obvious herniation and its sinus into the VSD), and severe (prolapse of the cusp and its sinus through the defect into the right ventricular [RV] outflow tract) (17, 18). AR was quantified using the ratio of jet width and LV outflow tract (LVOT) diameter. AR was classified as trivial (jet width/LVOT diameter < 10%), mild (jet width/LVOT diameter = 10–24%), moderate (jet width/LVOT diameter = 25–49%), and severe (jet width/LVOT diameter > 50%) (19).

The inclusion criteria were (1) the established diagnosis of dcVSD, (2) notably associated clinical symptoms (such as delayed growth, exercise intolerance, previous infectious endocarditis, recurrent respiratory infection, and congenital heart failure), (3) abnormal cardiac function on echocardiography (such as left ventricular overload and pulmonary artery hypertension), (4) heart murmur more than 2/6 grades and meanwhile unyielding request from the patient's guardian, (5) unobvious or mild AVP and AR, (6) defect diameter < 5 mm and (7) age ≥ 1 year and body weight ≥ 10 kg. Exclusion criteria were (1) moderate or severe AVP and AR, (2) significant comorbidities such as active infective endocarditis, contraindication to antiplatelet or anticoagulation therapy, and other congenital cardiac anomalies which need surgical treatment, etc., (3) severe pulmonary hypertension, with blood pressure higher than the aortic pressure (Eisenmanger syndrome), and the emergence of a bidirectional or right-to-left shunt, and (4) inability to provide informed consent.

### Device

The ADO-II (AGA Medical Corporation, MN, USA) has two retention flanges connected by a waist and consists of two layers of bare wire braid without fabric. The omission of the polyester layer of the earlier model of the ADO provides a lower entry profile, enabling delivery through a 4-French (F) or 5F delivery catheter (AGA Medical Corporation, MN, USA) with an internal diameter of 0.061 inches. Currently, the ADO-II is available with waist diameters of 3–6 mm and lengths of 4 and 6 mm.

### Procedure

The procedure was performed with patients under general anesthesia. Intravenous heparin (100 U/kg) was administered after the femoral venous and arterial access was established. After analysis of the hemodynamic data, left ventriculography and aortic root angiography were performed at the left oblique 80–100°/cranial 20° or at the right oblique 45° positions to assess the anatomy and size of the VSD, distance from the defect to the aortic valve and pulmonary valve, and severity of AVP and/or AR. An appropriate device was selected, and it was 1–2 mm larger than the defect measured by angiography.

**Figure 2:**
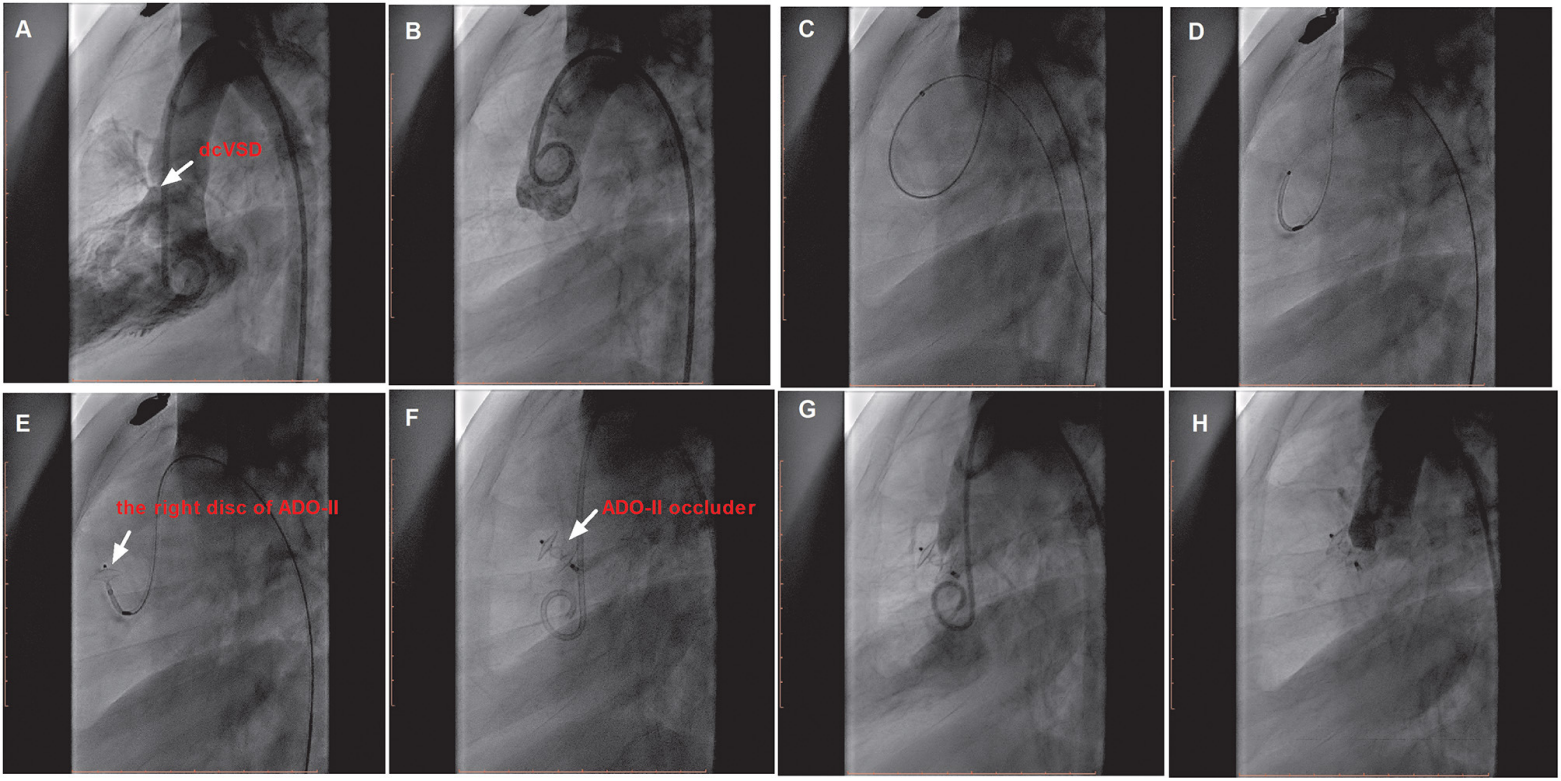
The procedure of transfemoral occlusion of the dcVSD using the ADO-II device *via* the retrograde approach. **(A)** Confirms the location of the deVSD (arrow) by left ventriculography before device closure of it, and **(B)** Demonstrates the finding of aortography without AR before the procedure. **(C–F)** Show the processes of the transcatheter technique. **(G)** Shows the operational success and no RS by left ventriculography, and **(H)** Shows no AR after device implantation by aortography. dcVSD, doubly committed subarterial ventricular septal defect; AR, aortic regurgitation; RS, residual shunt.

We preferentially attempted the retrograde approach because of the simplified manipulations (Figure 2). A 4 or 5 French cutoff pigtail catheter (SCW Medicath Ltd. Shenzhen, China) was carefully manipulated to pass through the defect into the right ventricle, and a 0.035/0.032-inch guide wire (Terumo, Tokyo, Japan) was advanced into the pulmonary artery or superior vena cava. Subsequently, the delivery sheath (AGA Medical Corporation, MN, USA) was replaced into the right ventricle. The selected device was retrogradely introduced through the delivery sheath. The right disk was pushed out of the sheath and then the delivery system was pulled back gently, finally releasing the waist and left disk until the waist and left disk were confirmed to be located on the left side of the septum. If the control angiography by hand from the sidearm of the delivery sheath and TTE showed satisfactory results without a residual shunt (RS) and new-onset AR, then the device was released.

**Figure 3:**
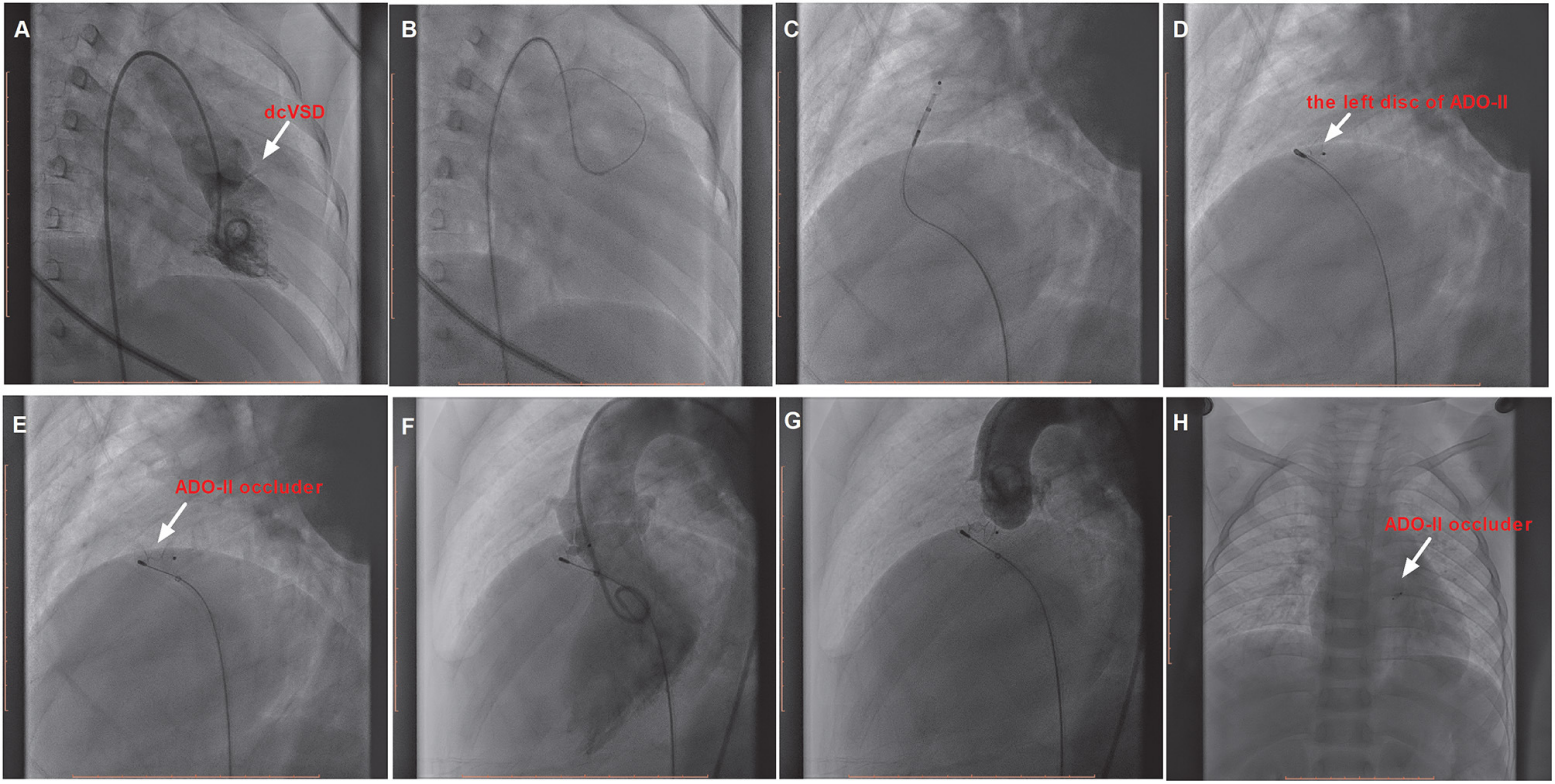
The procedure of transcatheter closure of the dcVSD using the ADO-II device *via* the antegrade approach. **(A)** Confirms the location of the dcVSD (arrow) with left-to-right shunting on left ventriculography before the procedure. **(B–E)** Show the whole processes of the transcatheter technique. **(F)** shows the operational success and no significant RS by left ventriculography, and **(G)** Shows no AR after device implantation by aortography before releasing the occluder. **(H)** Demonstrates the good position and configuration of the released occluder under X-ray fluoroscopy. dcVSD, doubly committed subarterial ventricular septal defect; AR, aortic regurgitation; RS, residual shunt.

However, if the delivery sheath was difficult to pass through the defect, the snare (Starway Medical, Beijing, China) was used *via* the femoral vein to entrap the wire and pull it outside the body in order to establish a femoral artery vein loop (arterialvenous loop). Then, the delivery sheath was advanced into the left ventricle through the arterialvenous loop and the device was deployed antegradely from the venous side (Figure 3). Both before deployment of the device and after releasing the device, repeated left ventricular and aortic root angiographies and TTE were performed to ensure that there was no RS and that the aortic valve was not affected.

### Follow-Up Protocol

All children undergoing successful VSD closure were kept on oral aspirin (3–5 mg/kg daily) for 6 months and underwent 72 h of dynamic electrocardiography (ECG) monitoring, and 12-lead ECG and echocardiography at 1, 3, and 7 days post-procedure. After discharge, patients were followed up clinically with 24 h dynamic ECG and echocardiography at 1, 3, 6, and 12 months during the first year and annually thereafter.

### Statistical Analysis

All analyses were performed with SPSS 17.0 (SPSS Inc.). The descriptive characteristics were presented as mean ± SD with range, and categorical variables as percentages. For the comparison of the differences between antegrade group and retrograde group in **Table 2**, the Mann–Whitney U test was used for the continuous variables and chi-square or Fisher's exact probability test for categorical variables. A *P* value < 0.05 was considered statistically significant.

## Results

The baseline demographic and clinical characteristics of all included patients are summarized in Table 1. Twenty-four children, including 17 male and 7 female patients, who underwent transfemoral occlusion of dcVSD using ADO-II were enrolled. Their median age was 3.2 years (1.6–12.6 years, 4.2 ± 3.1years) and their median weight was 13.3 kg (10.0–38.5 kg, 16.5 ± 7.7 kg). The median size of dcVSD detected by echocardiography was 3.0 mm (1.7–5.0 mm, 3.1 ± 0.8 mm). Without AR before the procedure, only mild aortic AVP was observed in one patient (case 12). As for the closure indications, the dcVSDs were closed in 5 children (cases 1, 4, 10, 13, and 19) due to refractory pneumonia and in 19 patients (cases 2, 3, 5, 6, 7, 8, 9, 11, 12, 14, 15, 16, 17, 18, 20, 21, 22, 23, and 24) because of a heart murmur more than 2/6 grades meanwhile plus unyielding request from the patient's guardian.

**Table 1:** The clinical characteristics of 24 patients with dcVSD.

**Before procedure**									**During procedure**				
**Case n**.	**Sex**	**Age** **(yr.)**	**BW (kg)**	**dcVSD size on echo. (mm)**	**Degree of AVP^**#**^**	**Degree** **of AR^**##**^**	**Associated diagnosis**	Indications for closure^*^	**Mean PA pressure (mmHg)**	**Defect diameter (mm)**	**Occluder size (mm)**	**Sheath size (F)**	**Antegrade or Retrograde**
1	M	2.0	10.0	2.5	None	None	–	a	22	2.0	3/4	4	Antegrade
2	M	1.6	12.0	3.0	None	None	–	c	26	3.0	3/4	5	Retrograde
3	M	1.8	12.0	2.5	None	None	–	c	23	2.0	3/4	4	Antegrade
4	M	3.1	13.0	3.5	None	None	–	a	21	2.0	4/4	4	Retrograde
5	M	3.1	13.0	3.0	None	None	–	c	17	1.5	3/4	4	Retrograde
6	F	3.1	13.5	3.0	None	None	–	c	20	2.0	3/4	4	Retrograde
7	M	3.6	14.0	3.5	None	None	–	c	23	2.0	3/4	4	Retrograde
8	M	5.2	17.0	4.0	None	None	–	c	25	2.0	3/4	4	Retrograde
9	F	8.6	21.0	3.0	None	None	–	c	25	2.5	5/4	5	Retrograde
10	F	6.0	27.0	4.0	None	None	–	a	24	2.7	5/4	5	Antegrade
11	F	12.6	36.0	4.0	None	None	–	c	21	2.0	3/4	4	Retrograde
12	M	8.3	24.0	5.0	Mild	None	–	c	28	2.0	4/4	5	Antegrade
13	M	6.8	18.0	1.5	None	None	–	a	21	1.5	3/4	4	Antegrade
14	M	2.0	10.5	3.0	None	None	–	c	19	2.0	3/4	4	Antegrade
15	F	2.0	10.2	2.0	None	None	–	c	17	1.5	3/4	4	Antegrade
16	M	3.3	14.0	1.7	None	None	–	c	18	2.0	3/4	4	Antegrade
17	M	4.2	15.0	4.0	None	None	–	c	20	2.5	3/4	4	Antegrade
18	M	2.6	13.0	4.0	None	None	–	c	20	3.3	4/4	5	Antegrade
19	F	2.5	13.5	3.0	None	None	–	a	17	1.5	3/4	4	Antegrade
20	M	1.6	11.0	3.0	None	None	ASD	c	25	2.0	3/4	4	Antegrade
21	M	3.6	13.0	2.0	None	None	–	c	22	1.5	3/4	4	Antegrade
22	M	4.6	12.5	3.0	None	None	–	c	20	1.5	3/4	4	Antegrade
23	M	2.3	13	3.5	None	None	–	c	20	3.5	4/4	5	Antegrade
24	F	10.5	38.5	2.5	None	None	–	c	25	2.5	4/4	4	Antegrade
**During procedure**						**After procedure**							
**Case n**.	**Operative time (min)**	**Fluoroscopic time (min)**		**Successful deployment**		**Hospital stay (days)**	**Postprocedure ECG**	**Follow–up duration** **(months)**		**Degree of residual shunt at least follow up**		**Degree of AR at least follow up**	**Other complication**
1	40	6.0		Yes		5	Normal	39+		–		–	None
2	75	17.0		Yes		4	Normal	40+		–		–	None
3	40	5.0		Yes		4	Normal	33+		Insignificant		–	None
4	30	3.0		Yes		5	Normal	41+		–		Mild	None
5	35	4.0		Yes		4	Normal	40+		–		–	None
6	60	5.0		Yes		4	Normal	39+		–		–	None
7	20	4.0		Yes		4	Normal	45+		–		–	None
8	52	5.0		Yes		4	Normal	38+		–		–	None
9	35	4.0		Yes		4	Normal	32+		–		–	None
10	40	5.0		Yes		4	Normal	32+		–		–	None
11	40	3.0		Yes		3	Normal	34+		–		–	None
12	75	–		No		2	–	–		–		–	–
13	45	6.5		Yes		3	Normal	27+		–		–	None
14	35	9.5		Yes		3	Normal	21+		–		–	None
15	40	6.0		Yes		3	Normal	20+		–		–	None
16	35	4.7		Yes		3	Normal	19+		–		–	None
17	37	6.0		Yes		3	Normal	18+		–		–	None
18	35	6.0		Yes		4	Normal	18+		–		–	None
19	26	3.0		Yes		4	Normal	29+		–		–	None
20	30	5.0		Yes		4	Normal	29+		–		–	None
21	30	5.0		Yes		5	Normal	17+		–		–	None
22	55	25		Yes		4	Normal	16+		–		–	None
23	50	10		Yes		3	Normal	2+		–		–	None
24	40	9		Yes		3	Normal	1+		–		–	None

*AR, aortic regurgitation; AVP, aortic valve prolapse; ASD, atrial septal defect; BW, body weight; dcVSD, doubly committed subarterial ventricular septal defect; echo., echocardiography; ECG, electrocardiography; F, female; M, male; PA, pulmonary artery; RS, residual shunt; yr., years*.

# ***Degree of AVP:***

*Mild AVP: buckling of the aortic cusp down the left ventricular (LV) outflow tract with minimal herniation into the VSD*.

*Moderate AVP: prolapse of the cusp with obvious herniation and its sinus into the VSD*.

*Severe AVP: prolapse of the cusp and its sinus through the defect into the right ventricular (RV) outflow tract*.

## ***Degree of AR:***

*Trivial AR: jet width/LVOT diameter < 10%*.

*Mild AR: jet width/LVOT diameter = 10–24%*.

*Moderate AR: jet width/LVOT diameter = 25–49%*.

*Severe AR: jet width/LVOT diameter > 50%*.

* ***Indications for closure:***

a *Notable clinical symptoms: refractory pneumonia, congestive heart failure, delayed growth, exercise intolerance, and previous infectious endocarditis*.

^b^*Signs of cardiac dysfunctions: left ventricular overload, pulmonary hypertension, or mild aortic valve prolapse on echocardiography that slight downward displacement of the coaptation point of the aortic cusps lead to straightening of the cusps in diastole*.

c *Heart murmur more than 2/6 grades. The background color in the line of case 12 is to highlight the only one failed case*.

As for the procedure-associated parameters, the median pulmonary artery (PA) pressure was 21.0 mmHg (17.0–28.0 mmHg, 21.6 ± 3.1 mmHg). The median diameter of the dcVSD detected by angiography was 2.0 mm (1.5–3.5 mm, 2.1 ± 0.6 mm). The device diameter was 3/4 mm in 17 cases (70.8%), 4/4 mm in 5 cases (20.8%), and 5/4 mm in 2 cases (8.3%), and the delivery sheaths were 4F in 18 cases (75.0%), and 5F in 6 cases (25.0%). In total, 16 patients were closed by the antegrade approach (66.7%) and 8 patients by the retrograde approach (33.3%); both approaches had no significant difference except for the notably lower fluoroscopic time with the retrograde approach than with the antegrade approach (*P* < 0.05) (the comparison of both approaches is shown in Table 2). The median operative duration was 40.0 min (20.0–75.0 min, 41.7 ± 13.7 min), and the median fluoroscopic time was 5.0 min (3.0–25.0 min, 6.8 ± 5.0 min). Overall, the device was implanted successfully in 95.8% of the patients (23/24).

**Table 2:** Comparison of patient characteristics between using the antegrade approach and using the retrograde approach.

**Parameters**	**Antegrade approach (*n* = 16)**	**Retrograde approach (*n* = 8)**	***P*** **value^**#**^**
**Baselines**			
Age (years)	3.0 (1.6–10.5, 4.0 ± 2.6)	3.4 (1.6–12.6, 5.1 ± 3.7)	0.208
Sex, *n* (%)			
Male	12 (75.0%)	5 (62.5%)	0.428
Female	4 (25.0%)	3 (37.5%)	
Weight (kg)	13.0 (10.0–38.5, 16.0 ± 7.7)	13.8 (12.0–36.0, 17.4 ± 8.1)	0.340
dcVSD size on echo. (mm)	3.0 (1.5–5.0, 3.0 ± 1.0)	3.3 (3.0–4.0, 3.4 ± 0.4)	0.167
Cardiac complications before the procedure, *n* (%)			
mild AVP	1 (6.3%)	0	–
mild AR	0	0	–
ASD	1 (6.3%)	0	–
^*^Indications for closure, *n* (%)			
^a^ Notable clinical symptoms	4 (25.0%)	1 (%)	–
^b^ Signs of cardiac dysfunctions	0 (0.0%)	0 (0.0%)	–
^c^ Heart murmur	12 (75.0%)	7 (%)	–
**Procedure**			
Mean PA pressure (mmHg)	20.5 (17.0–28.0, 21.3 ± 3.1)	22.0 (17.0–26.0, 22.3 ± 3.1)	0.370
Diameter of the defect (mm)	2.0 (1.5–3.5, 2.1 ± 0.6)	2.0 (1.5–3.0, 2.1 ± 0.4)	0.770
Operative time (min)	40.0 (26.0–75.0, 40.8 ± 11.7)	37.5 (20.0–75.0, 43.4 ± 17.8)	0.951
Fluoroscopic time (min)	6.0 (3.0–25.0, 7.5 ± 5.2)	4.0 (3.0–17.0, 5.6 ± 4.7)	0.026^*^
Size of the occluder, *n* (%)			
3/4 mm	11 (68.7%)	6 (75.0%)	–
4/4 mm	4 (25.0%)	1 (12.5%)	–
5/4 mm	1 (6.3%)	1 (12.5%)	–
Sheath size, *n* (%)			
4F	12 (75.0%)	6 (75.0%)	–
5F	4 (25.0%)	2 (25.0%)	–
Device implantation success, *n* (%)	15 (93.8%)	8 (100%)	0.667
**Postprocedure**			
Hospital stay (days)	4.0 (2.0–5.0, 3.6 ± 0.8)	4.0 (3.0–5.0, 4.0 ± 0.5)	0.150
Abnormal postoperative ECG	0	0	–
**Follow up**			
Follow-up duration (months)	1+ to 39+	32+ to 45+	–
Complications associated with the procedure, *n* (%)			
mild AR	0	1 (12.5%)	–
mild RS	1 (6.3%)	0	–
Others such as LVOT	0	0	–
None	15 (93.8%)	7 (87.5%)	0.565

*ASD, atrial septal defect; AR, aortic regurgitation; AVP, aortic valve prolapses; dcVSD, doubly committed subarterial ventricular septal defect; ECG, electrocardiography; echo., echocardiography; F, French; LVOT, left ventricular outflow tract obstruction; PA, pulmonary artery; RS, residual shunt*.

# *Mann-Whitney U test was used for the continuous variables, and Chi-square or Fisher's exact probability test for categorical variables*.

* *Statistically significant (P < 0.05)*.

*Data are expressed as median (range, mean ± SD) or n (%). The color is to separate from the different parts*.

**Figure 4:**
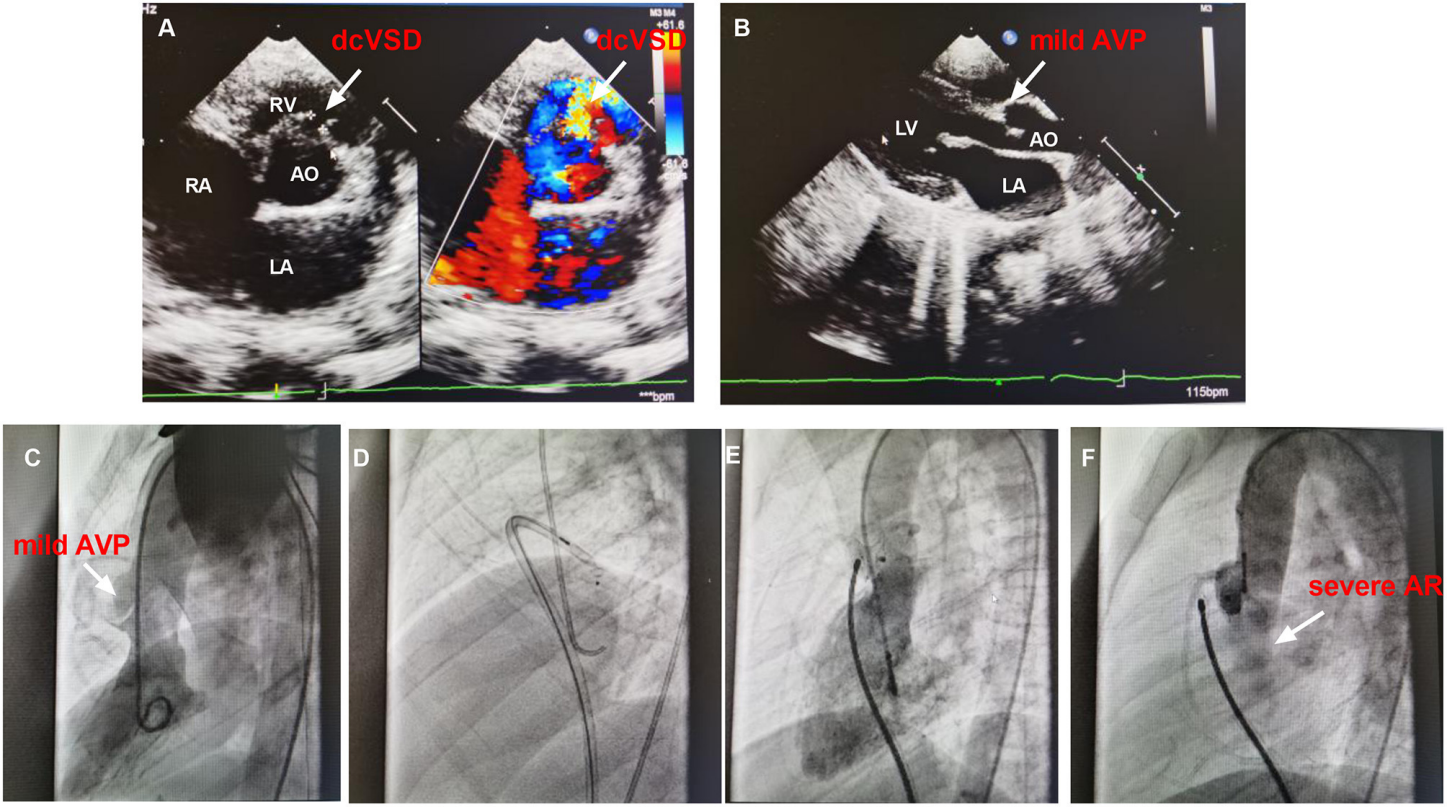
The images of TTE and angiography of case 12. **(A,B)** shows the dcVSD and the mild AVP on TTE. **(C)** Shows the mild AVP by left ventricular angiography. **(D)** Demonstrates the closure using ADO-II and **(E)** Shows the closure with symmetrical double-disk occluder. **(F)** Shows the AR after deploying occluder. AO, aorta; AR, aortic regurgitation; AVP, aortic valve prolapse; dcVSD, doubly committed subarterial ventricular septal defect; LA, left atrium; LV, left ventricle; RA, right atrium; RV, right ventricle; TTE, transthoracic echocardiography.

The failed case 12 was an 8.3-year-old male weighing 24.0 kg with a complaint of heart murmur of 3/6 grades. TTE before closure revealed a 5.0 mm sized dcVSD with a left to right shunt, mild AVP, and left atrium enlargement (25 mm) (Figures 4A,B). The defect measured 2.0 mm on left ventricular angiography and mild to moderate AVP was observed (Figure 4C). A 4/4 mm sized ADO-II was initially chosen (Figure 4D). After the establishment of the arterial–venous loop, the delivery sheath was advanced into the LV. First, the left disk was pushed out of the sheath and then the delivery system was pulled back gently. However, when we tried to release the waist and right disk of the device, both the delivery system and the occluder slid to the right ventricle immediately and easily. Thereafter, we re-analyzed the left ventricular angiogram and found that the size of dcVSD was greatly underestimated because of the AVP. The size of the defect was re-measured as large as 8.0 mm. A symmetrical double-disk occluder (SHAMA, Shanghai, China) sized 10 mm was used to close the defect (Figure 4E). However, before the deployment of the occluder, both the aortic root angiography and TTE revealed moderate to severe AR (Figure 4F). At last, the occluder was withdrawn and the patient was transferred to the cardiac surgery department in West China hospital for surgical closure of dcVSD.

All 23 patients who successfully received device closure had a normal postoperative ECG. The median hospital stay was 4.0 days (2.0–5.0 days, 3.7 ± 0.8 days). During the follow-up of 1+ to 45+ months, there were two cases with complications (2/23, 8.7%): mild AR was observed in case 4 and insignificant RS was detected in case 3 as shown in Table 1. Left ventricular outflow tract obstruction, atrioventricular block, and significant arrhythmia were not noted. The overall characteristics of the enrolled patients are summarized in supplemental Supplementary Table 1.

## Discussion

To our best knowledge, this is the largest cohort with short-/mid-term follow-up results for transfemoral occlusion of small dcVSD using ADO-II device in children. In this study, we proved that this technique was associated with a relatively high success rate (95.8% among the selected population) and multiple advantages, including 1) fewer restrictions by body weight and patient age, 2) simpler deployment manipulations with either antegrade or retrograde approach, 3) shorter procedure time and fluoroscopic time, 4) no major complications, and 5) low rate of RS and new-onset aortic valve complications. Most importantly, we suggested that transfemoral occlusion of dcVSD using ADO-II should be applied in small defects <5.0 mm without AVP/AR.

Previously, Chen et al. (8) recruited 50 children with dcVSD who underwent transfemoral occlusion with an asymmetric occluder to compare the mid-term outcomes of transfemoral and perventricular device occlusions and conventional surgical closure. They also found that the procedure success rate was far lower (66% [33/50]) than that of the other groups. The poor success rate might be attributable to the special anatomy of the dcVSD and the features of the selected asymmetric device. The dcVSD is located in the infundibular septum and close to the pulmonary valve and aortic valve annulus. Several technical challenges for transcatheter closure of dcVSD can be encountered including the difficult establishment of the arteriovenous wire loop, difficult advancement of the wire loop and delivery sheath to the left ventricular apex, difficult adjustment of the position and orientation of the device because of the acute angle between the delivery system and dcVSD, and the possible underestimation of the defect size because of the partial coverage by the right coronary cusp (8). Besides, based on the special anatomy of the dcVSD, the characteristics of the asymmetric device might have partially contributed to the failure, suggesting the necessity of the appropriate device choice for transcatheter closure of dcVSD.

Afterward, one study conducted by Shyu et al. (9) enrolled nine patients with supracristal VSD who underwent transfemoral closure using ADO-I with a success rate of 78% (7/9). The reasons for failure were an excessively large defect in one and device-induced significant AR in the other case (the only included child aged 3.0 years). During the follow-up, development or worsening of AR was observed in two patients at 6 and 12 months. Subsequently, Huang et al. (10) reported 22 patients (including 12 children) with dcVSD who received transfemoral closure using ADO-I with a success rate of 95.4% (21/22). But device-induced mild AR was found in nine (42.8%) patients post-procedurally and during their in-hospital stay; the condition was resolved in two (9.5%) patients and unchanged in seven (33.3%) patients at the last follow-up. Another four (19.0%) patients newly developed mild AR during the follow-up. Collectively, despite the relatively high success rate achieved using ADO-I, new-onset or worsening of AR was still the main issue which may be attributable to the sharp and relatively stiff edge of the left disc of the ADO-I and a weaker aortic valve structure in the younger patients compared with the older patients.

Besides, Konar multifunctional occluder (MFO; Lifetech Scientific Corporation, Shenzhen, China) and Nit Occlud Le^*VSD*^ Coil (Produkte fur die Medizin/PFM, Koln, Germany) have also been used to close dcVSD in recent years. Kuswiyanto et al. (20) reported the efficacy and outcome of transcatheter closure of dcVSD with various types of devices. In the 40 patients where the device was attempted, MFO and Le VSD coil were used in 13 and 8 patients, respectively. They found that the left disc of MFO is stiffer and it may still interfere with the aortic valve resulting in AR since the only patient who developed new-onset AR in their series was closed with MFO. As for the Le VSD coil, mechanical hemolysis still remains the issue of concern because of a tendency of a more residual shunt. The absence of a self-expandable characteristic leading to a “stent” effect into the defect could explain the tendency of a higher rate of the residual shunt with this device. In their series, one patient experienced persistent severe hemolysis on the 10th day after discharge related to the residual shunt through the coil and required multiple blood transfusions despite conservative treatment.

Compared with MFO and Le VSD coil, the ADO-II occluder is softer and more flexible and has been proven to be preferable to closing the defects of moderate size (2–5 mm), especially in infants and small children (11). The ADO-II can be implanted either through the antegrade approach or *via* the retrograde approach with simpler manipulations. It easily bends to the plane of the aortic valve without distorting the coaptation mechanism. Our study agreed with Rahmat Budi Kuswiyanto's and Lin's studies (14, 20) that the use of ADO-II device achieved a high success rate and low incidence of device-related complications to close outlet-type VSD both in children and adults.

However, there is a lack of evidence-based indications or contraindications for preoperative patient selection about the new technique. According to our experience, the patient selection criteria for transfemoral dcVSD occlusion using ADO-II are proposed as follows: first, procedure-induced aortic valve dysfunction is a key safety factor needing to be considered because of the close relationship between the device and the valve. The interface between the device and the aortic cups may be still unavoidable due to the dynamic motion of the aortic valve and subsequently cause aortic valve injury and aortic insufficiency during mid-/long-term follow-up even after total endothelialization of the device. Besides, AVP could increase the risk of the interface between the aortic valve and device. The higher incidence of postprocedural AR, as reported in Lin HC's study (14), was likely to partly result from the pre-existing mild AVP. Also, AVP may result in the underestimation of defect size and thereby increase the risk of procedure failure. In our study, the procedure failure for case 12 resulted from the underestimation of defect size due to the preoperative AVP. A previous study (21) also demonstrated the association of procedure failure with the occurrence of preoperative AVP (even of mild degree). Similar findings were also observed in Kuswiyanto' (20). Failure to attempt occurred in two patients with pre-existing AVP and AR due to undersize of the defect and one patient showed moderate residual shunt and worsened AR after device deployment. Therefore, we consider that pre-existing AVP or AR, even in a mild degree may indicate a higher risk of procedure failure or worsening the degree of regurgitation and can be made as exclusion criteria for transcatheter closure. Second, although the relatively large dcVSD are easier to cross, they are more prone to dislodge because of an insufficient amount of tissue around the entire circumference of the dcVSD. Also, using a larger occluder may contribute to device-related aortic insufficiency. As for a small dcVSD, the aortic and pulmonary rim may be deficient, but the rest of the rim could still enhance the stability of the occluder in dcVSD owing to the small size and softness of ADO-II. Therefore, this technique may be suitable for selected patients with a small dcVSD of <5.0 mm. At last, several previous studies have proven that ADO-II or the additional size device of ADO-II was suitable for small children and infants including newborns (22–24), suggesting that this technique may be less restricted by age and body weight. But, the lowest weight was 10.0 kg and the youngest age was 1.6 years in our study. Based on the limited samples in this study, we thought that this procedure should be performed in those older than 1 year of age because infants probably have a weaker aortic valve structure and the risk of device-induced AR postoperatively may be higher.

Furthermore, regarding the indications for closure, most of the patients in this series are small dcVSD without significant hemodynamic changes. Except for the patient preference or social pressures as well as primary prophylaxis of the risk of bacterial endocarditis, the main reason for early closure of dcVSD is to avoid the further associated complication of cusp prolapse and valve regurgitation. Unlike the perimembranous and muscular VSD, spontaneous closure of dcVSD is rare and subsequent complications, mainly aortic valve deformity, are relatively frequent and easily progressive. The incidence of AVP and AR in dcVSD in the literature is reported to be 43–73% and 24–65, respectively. In Lun et al. (25) study, in which they enrolled 214 patients with dcVSD, of the 139 asymptomatic patients managed conservatively, 102 (73%) developed AVP, 78% of whom (80/102) developed AR. The prevalence of AVP and AR were gradually increased for 8, 30, 64, and 83%, and 3, 24, 45, and 64% at 1, 5, 10, and 15 years old, respectively. Furthermore, pre-existing AVP or AR, even to a mild degree may indicate a higher risk of procedure failure or worsening the degree of regurgitation. The incidence of AR post-surgical closure was also higher, ranging between 28 and 63%. The degree of AR appeared to persist and similarly even after surgical closure of dcVSD (20). Therefore, it is generally accepted that dcVSD should be early closed irrespective of cusp prolapse or valve regurgitation, even in regions with low resources.

### Limitations

Our study had some limitations, including the small samples and lack of long-term follow-up. Transfemoral occlusion of small dcVSD using ADO-II in this study was primarily based on our experience as opposed to a large randomized controlled trial. Despite these limitations, we demonstrated the feasibility and safety for transfemoral occlusion of small dcVSD using ADO-II and provided an alternative approach to close small dcVSD in children. Prior to the operation, the indications must be rigorously evaluated as suggested earlier. In addition, this procedure should be performed at a medical center with extensive experience of transfemoral occlusion of VSDs. Besides, long-term follow-up studies on a larger number of patients are essential to assess the natural course of the post-transfemoral device occlusion structure inside the heart and further prove the safety and feasibility of transfemoral closure of small dcVSD using ADO-II.

## Conclusion

Taken together, it should be emphasized that surgical repair is irreplaceable in many situations and percutaneous perventricular device closure can achieve acceptable outcomes in selected patients with dcVSD. However, transfemoral occlusion may be an alternative method to avoid negative events related to invasive surgery. According to our initial experience, transfemoral occlusion of small dcVSD <5.0 mm without AVP/AR using ADO-II was technically feasible and safe in selected children with acceptable short-/mid-term outcomes, but development or worsening of AR requires long-term follow-up.

## Data Availability Statement

The original contributions presented in the study are included in the article/Supplementary Material, further inquiries can be directed to the corresponding author/s.

## Ethics Statement

This study was approved by the University Ethics Committee on Human Subjects at Sichuan University [No. 2015(010)]. Written informed consent to participate in this study was provided by the participants' legal guardian/next of kin. Written informed consent was obtained from the individual(s), and minor(s)' legal guardian/next of kin, for the publication of any potentially identifiable images or data included in this article.

## Author Contributions

CW, YH, KZ, and YL: conceptualization. CT, KZ, and XL: resources. CT, CW, and SS: formal analysis and investigation. CW, YH, KZ, and XL: funding acquisition. CT and KZ: writing-original draft preparation. YH and CW: writing-review and editing. All authors contributed to the article and approved the submitted version.

## Funding

This study was supported by grants from the National Key R&D Program of China (No. 2018YFC1002301), National Natural Science Foundation of China (Nos. 81800288 and 81971457), and Science-technology Support Plan Projects in Sichuan province (Nos. 2019YFS0243 and 2020YFS0101). This study was also supported by the Fundamental Research Funds for the Central Universities.

## Conflict of Interest

The authors declare that the research was conducted in the absence of any commercial or financial relationships that could be construed as a potential conflict of interest.

## Publisher's Note

All claims expressed in this article are solely those of the authors and do not necessarily represent those of their affiliated organizations, or those of the publisher, the editors and the reviewers. Any product that may be evaluated in this article, or claim that may be made by its manufacturer, is not guaranteed or endorsed by the publisher.
