# Supplementary material for: Transfemoral Occlusion of Doubly Committed Subarterial Ventricular Septal Defect Using the Amplatzer Duct Occluder-II in Children
Source: Front Cardiovasc Med. 2022 Apr 12;9:837847. doi: 10.3389/fcvm.2022.837847 (PMC9039183; doi:10.3389/fcvm.2022.837847)
Supplement: Supplementary file 1 [file Table_1.DOCX]

Supplemental Table S1 Overall characteristics of the 24 patients

| **Parameters** | **Data [Mean ± SD (range)]** |
| --- | --- |
| Baselines |  |
| Age (years) | 4.2 ± 3.1 (1.6-12.6) |
| Sex, n (%) | |
| Male | 17 (70.8%) |
| Female | 7 (29.2%) |
| Body Weight (kg) | 16.5 ± 7.7 (10.0-38.5) |
| dcVSD size on echo. (mm) | 3.1 ± 0.8 (1.7-5.0) |
| Preprocedural AVP or AR |  |
| mild AVP | 1 (4.2%) |
| mild AR | 0 |
| Associated diagnosis |  |
| ASD | 1 (4.2%) |
| ^*^Indications for closure, n (%) | |
| ^a^ Notable clinical symptoms | 5 (20.8%) |
| ^b^ Signs of cardiac dysfunctions | 0 |
| ^c^ Unyielding parental requests | 19 (79.2%) |
| Procedure | |
| Mean PA pressure (mmHg) | 21.6 ± 3.1 (17.0-28.0) |
| Diameter of the defect (mm) | 2.1 ± 0.6 (1.5-3.5) |
| Operative time (min) | 41.7 ± 13.7 (20.0-75.0) |
| Fluoroscopic time (min) | 6.8 ± 5.0 (3.0-25.0) |
| Size of the occulder, n (%) | |
| 3/4 mm | 17 (70.8%) |
| 4/4 mm | 5 (20.8%) |
| 5/4 mm | 2 (8.3%) |
| Sheath size, n (%) |  |
| 4F | 18 (75.0%) |
| 5F | 6 (25.0%) |
| Approach of closure, n (%) | |
| Antegrade | 16 (66.7%) |
| Retrograde | 8 (33.3%) |
| Device implantation success, n (%) | 23 (95.8%) |
| Postprocedure and follow-up (n=23) | |
| Hospital stay (days) | 3.7 ± 0.8 (2.0-5.0) |
| Postoperative ECG, n (%) |  |
| Normal | 23 (100.0%) |
| Follow-up duration (months) | 1+ to 45+ |
| Complications associated with the procedure, n (%) | |
| mild AR | 1 (4.3%) |
| mild RS | 1 (4.3%) |
| Others such as LVOT | 0 |

ASD: atrial septal defect; AR: aortic regurgitation; AVP: aortic valve prolapse; dcVSD: doubly committed subarterial ventricular septal defect; ECG: electrocardiography; Echo.: echocardiography; F: French; LVOT: left ventricular outflow tract obstruction; PA: pulmonary artery

**^*^ Indications for closure:**

a. Notable clinical symptoms: refractory pneumonia, congestive heart failure, delayed growth, exercise intolerance, and previous infectious endocarditis.

b. Signs of cardiac dysfunctions: left ventricular overload, pulmonary hypertension, or mild aortic valve prolapse on echocardiography.

c. Heart murmur more than 2/6 grades.
